# Clinical Impact of Delayed Initiation of Adjuvant Chemotherapy Among Patients With Stage II/III Gastric Cancer: Can We Do Better?

**DOI:** 10.3389/fonc.2020.01149

**Published:** 2020-07-31

**Authors:** Qi-Yue Chen, Zhi-Yu Liu, Qing Zhong, Jian-Wei Xie, Jia-Bin Wang, Jian-Xian Lin, Jun Lu, Long-Long Cao, Mi Lin, Ru-Hong Tu, Ze-Ning Huang, Ju-Li Lin, Hua-Long Zheng, Ping Li, Chao-Hui Zheng, Chang-Ming Huang

**Affiliations:** ^1^Department of Gastric Surgery, Fujian Medical University Union Hospital, Fuzhou, China; ^2^Department of General Surgery, Fujian Medical University Union Hospital, Fuzhou, China; ^3^Key Laboratory of Ministry of Education of Gastrointestinal Cancer, Fujian Medical University, Fuzhou, China; ^4^Fujian Key Laboratory of Tumor Microbiology, Fujian Medical University, Fuzhou, China

**Keywords:** gastric carcinoma, prognosis, untimely, chemotherapy, risk factor

## Abstract

**Background:** To investigate the prognostic effects and risk factors of the omission and delay of postoperative chemotherapy of stage II/III gastric cancer (GC).

**Methods:** The clinicopathological data of 1,520 patients undergoing radical gastrectomy for stage II/III GC were collected and retrospectively analyzed. We defined the chemotherapy delayed until more than 60 days after radical gastrectomy and the complete omission of chemotherapy as unacceptable chemotherapy initiation (UAC), whereas the chemotherapy conducted within 60 days of radical gastrectomy was defined as acceptable chemotherapy initiation (AC). The survival between the two groups was compared, and the trends and risk factors of UAC were analyzed.

**Results:** There were 539 (35.5%) patients with UAC. The overall survival (OS) and disease-free survival of the UAC group patients were significantly inferior to those in the AC group (*p* < 0.001). Cox multivariate analysis demonstrated that UAC is an independent predictor of OS (*p* < 0.05). The OS and disease-free survival of the patients in the UAC group were close to those of the patients without chemotherapy (*p* > 0.05). Logistic analysis showed that female, old age, a self-paid status, a very low social status, high American Society of Anesthesiologists scores, intra-abdominal surgery history, and serious postoperative complications were independent risk factors of UAC (all *p* < 0.05). The radar chart shows the risk factors of UAC changed with time.

**Conclusions:** UAC after radical gastrectomy is an independent risk factor for the prognosis of stage II/III GC patients. However, no significant decline of UAC has been achieved recently and should call for the attention of both government and clinicians.

## Introduction

Although the incidence of gastric cancer (GC) has declined in recent years, its mortality rate remains at the forefront of cancer-related deaths (1–3). Scholars have spent great effort to explore how to improve the survival rate of GC, particularly in advanced-stage patients, including the constant perfection of surgical methods and the continuous improvement of chemotherapy regimens (4–10); however, the effect is still not ideal. Therefore, it may not be enough to rely solely on clinicians to improve the overall survival (OS) rate of GC. The concerted efforts of the government, the family, and the patients should be used. In particular, for the majority of Chinese patients with locally advanced GC (stage II/III) (11), the regulation of D2 radical surgery may only be the beginning of treatment. Whether adjuvant chemotherapy can be performed in time may also determine the overall effect of treatment. We observed in clinical practice that some patients with delayed chemotherapy had a significantly poorer prognosis compared with those with timely chemotherapy. The delay or omission of chemotherapy may occur for various reasons in patients with GC, particularly in developing countries, such as China. It is critical for clinical workers and health departments to determine its precise effect on prognosis and how to detect high-risk patients as early as possible. However, to our knowledge, although several studies have reported the chemotherapy time impacting on the prognosis of patients with GC (12–15), the understanding of omission and delay of chemotherapy is still controversial. Closure of this knowledge gap is critical for researchers, surgeons, and administrators. Clearly, it is unfeasible and unethical to explore this issue through prospective trials. Therefore, this paper aims to investigate the effects of the omission and delay of chemotherapy in stage II/III GC and its risk factors in recent years from a large tertiary referral center in southern China; moreover, the goals are to assess the effect on prognosis and identify the high-risk factors of delayed chemotherapy and the trends in recent years to provide a reference for the intervention of relevant departments, including the government and clinicians.

## Materials and Methods

### Study Design and Patients

In this study, the clinical and pathological data of 2,604 patients with GC at the Fujian Medical University Union Hospital (FMUUH) from January 2011 to April 2015 were retrospectively analyzed. The inclusion criteria were as follows: (1) Preoperative endoscopic biopsy-confirmed GC; (2) Receiving a D2 lymph node dissection surgery; and (3) Radical surgery. Furthermore, the case exclusion criteria were as follows: (1) GC was confirmed as stage I or IV by the postoperative pathology (*n* = 1,025 cases); (2) Histological identification of a tumor type other than adenocarcinoma (*n* = 13 cases); (3) Remnant GC (*n* = 23 cases); and (4) Survival time is <3 months (*n* = 23 cases). Finally, 1,520 patients with stage II/III gastric adenocarcinoma treated with D2 radical resection were included. Supplementary Figure 1 shows the screening process. This study was conducted with the approval of the institutional review boards at FMUUH. The time of chemotherapy was defined as the interval between the radical gastrectomy and the first initiation time of chemotherapy. According to the correlation between the OS rate and the chemotherapy time, the cutoff value of the chemotherapy time was selected (Supplementary Figure 2). The results showed that the OS of the patients treated with chemotherapy within <4 weeks, 4–6 weeks, and 6–8 weeks were significantly better than those who had no chemotherapy (*p* < 0.05), whereas the OS of the patients treated with chemotherapy after 8–10 weeks, 10–12 weeks, and more than 12 weeks were similar to those without chemotherapy (*p* > 0.05). To facilitate the analysis, we selected the cutoff point with the duration of chemotherapy of more than 60 days as the delay of chemotherapy.

Accordingly, we defined chemotherapy that was delayed until more than 60 days after radical gastrectomy and the complete omission of chemotherapy as unacceptable chemotherapy initiation (UAC group), whereas chemotherapy conducted within 60 days of radical gastrectomy was defined as acceptable chemotherapy initiation (the AC group). The chemotherapy regimen did not change for each patient unless there was a severe chemotherapy reaction. This retrospective study was approved and implemented by the Ethics Committee of Fujian Medical University Union Hospital.

### Methods

Preoperative imaging studies were routinely performed after endoscopic and upper gastrointestinal examinations with contrast to confirm the tumor location and included chest radiography, computed tomography (CT) scanning, ultrasonography (US) of the abdomen and bone scanning, and positron emission tomography–computed tomography (PET–CT) as required to evaluate the clinical stage. We used CT scans and the seventh edition of the International Union Against Cancer (UICC) classification system to assess the clinical and pathologic stage (16). According to the 2014 version of the *Japanese Gastric Cancer Treatment Guidelines* (17), our center recommended 5-Fu-based chemotherapy for patients with postoperative pathological stage II/III. The patient's residential address, marital status, procreation status, type of medical insurance, occupation, smoking history, alcohol consumption, and other information were routinely recorded in the electronic database of the Fujian Medical University Union Hospital medical records. The financial condition of the patient was recorded by the health care system. We conducted a comprehensive assessment of the patient's social status based on the patient's occupation, residential address, education, and economic conditions, according to literature (18).

### Follow-Up

The last follow-up time was April 2018. The follow-up rate of the 1,520 patients was 95.9%. Postoperative follow-ups using outpatient, hospitalization, etc. were every 3 months for the first 2 years, every 6 months for 3–5 years, and every year for after 5 years. The overwhelming majority of the patients routinely received physical examinations, laboratory tests (including carbohydrate antigen 19-9, cancer antigen 72-4, and carcinoembryonic antigen levels), chest films, full belly color Doppler ultrasound or abdominal CT, and an annual gastroscopy. The OS time represents the time from the operation to the last follow-up or death. Disease-free survival (DFS) was defined as the time from the surgery to the time of recurrence or death from any other cause.

### Statistical Analysis

All data were processed using SPSS 20.0 (SPSS Inc., Chicago, IL). Continuous variables were analyzed with Student's *t*-tests, and categorical variables were analyzed with χ2 or Fisher's tests. The survival rate was calculated using the Kaplan–Meier method, and the survival rates were compared with the Log-rank test. The risk factors related to UAC were analyzed using a logistic model, with a Cox proportional risk model for multivariate prognostic analysis. Stepwise backward variable removal was applied to the multivariate model to identify the most accurate and parsimonious set of predictors. A *p*-value of less than 0.05 was considered statistically significant.

## Results

### Patient Characteristics Between Unacceptable Chemotherapy Initiation and Acceptable Chemotherapy Initiation Groups

Of all patients, there were 539 patients with UAC, with an incidence of 35.5%. Table 1 shows the clinicopathological data of the patients in the AC and UAC groups. There were significant differences in terms of age, sex, medical insurance type, income, social status, residential address, American Society of Anesthesiologists (ASA) score, intra-abdominal surgery history, comorbidities, Charlson score, surgery period, and postoperative complications between the two groups (*p* < 0.05); however, the body mass index (BMI), occupation, marital status, procreation status, smoking and drinking consumption, abdominal surgery history, tumor site, pathological tumor stage, pathological node stage, pathological tumor–node–metastasis (pTNM) staging, tumor size, number of harvested lymph nodes, lymphatic vessel infiltration, and pathological differentiation degree were not significantly different between the groups (*p* > 0.05). The median follow-up time for the AC and UAC groups were 43 (3–86) months and 37 (3–91) months, respectively.

**Table 1:** Sociodemographic and clinicopathologic variables of the AC and UAC groups.

**Variable**	**AC group (*****n*** **=** **981)**		**UAC group (*****n*** **=** **539)**		***P***
	**No. of patients**	**%**	**No. of patients**	**%**	
**Sex**					**0.030**
Female	234	23.9	156	28.9	
Male	747	76.1	383	71.1	
**Age, year**					**<0.001**
<65	669	68.2	234	43.4	
≥65	312	31.8	305	56.6	
**BMI, kg/m**^**2**^					0.470
<18.5	107	10.9	70	13	
18.5–24.9	732	74.6	395	73.3	
≥25.0	142	14.5	74	13.7	
**Medical insurance type**					**<0.001**
Self-paid	69	7.0	83	15.4	
Rural insurance	569	58.0	262	48.6	
Urban insurance	337	34.4	191	35.4	
Others	6	0.6	3	0.6	
**Occupation**					0.533
No	937	95.5	511	94.8	
Yes	44	4.5	28	5.2	
**Income**					**0.004**
Very low	176	17.9	136	25.2	
Low	602	61.4	296	54.9	
General	173	17.6	97	18.0	
High	30	3.1	10	1.9	
**Social status**					**<0.001**
Very low	5	.5	15	2.8	
Low	208	21.2	177	32.8	
General	686	69.9	297	55.1	
High	65	6.6	44	8.2	
Very high	17	1.7	6	1.1	
**Residential address**					**0.011**
Village	615	62.7	302	56.0	
City	366	37.3	237	44.0	
**Marital status**					0.542
No	11	1.1	8	1.5	
Yes	970	98.9	531	98.5	
**Procreation status**					0.799
No	13	1.3	8	1.5	
Yes	968	98.7	531	98.5	
**Smoking and drinking consumption**					0.211
No	582	59.3	344	63.8	
Smoking	229	23.3	114	21.2	
Drinking	31	3.2	15	2.8	
Both	131	13.4	56	10.4	
**ASA score**					**<0.001**
I	622	63.4	227	51.4	
II	328	33.4	228	42.3	
III–IV	31	3.2	34	6.3	
**Abdominal surgery history**					**<0.001**
No	876	89.3	439	81.4	
Yes	105	10.7	100	18.6	
**Intra-abdominal surgery history**					0.500
No	912	93.0	496	92.0	
Yes	69	7.0	43	8.0	
**Comorbidity**					**<0.001**
No	730	74.4	345	64.0	
Yes	251	25.6	194	36.0	
**Charlson score**					**<0.001**
0	730	74.4	345	64.0	
1–2	236	24.1	187	34.7	
3–5	15	1.5	7	1.3	
**Tumor site**					0.684
Lower	352	35.9	194	36.0	
Middle	223	22.7	109	20.2	
Upper	272	27.7	157	29.1	
Overlapping lesion of stomach	134	13.7	79	14.7	
**Surgery period**					**<0.001**
2011	162	16.50	184	34.10	
2012	263	26.80	122	22.60	
2013	241	24.60	102	18.90	
2014	254	25.90	104	19.30	
2015	61	6.20	27	5.00	
**Depth of invasion (pT)**					0.296
Mucosa/Submucosa	24	2.4	9	1.7	
Proper muscle	82	8.4	33	6.1	
Subserosa	421	42.9	243	45.1	
Serosa	454	46.3	254	47.1	
**Nodal status (pN)**					0.284
N0	140	14.3	92	17.1	
N1	176	17.9	82	15.2	
N2	246	25.1	127	23.6	
N3	419	42.7	238	44.2	
**pTNM stage**					0.222
IIA	153	15.6	90	16.7	
IIB	159	16.2	80	14.8	
IIIA	171	17.4	72	13.4	
IIIB	234	23.9	144	26.7	
IIIC	264	26.9	153	28.4	
**Tumor size, mm**					0.105
<20	31	3.2	17	3.2	
20–50	546	55.7	270	50.1	
>50	404	41.2	252	46.8	
**Examined LNs, no**.					0.629
≤15	25	2.5	16	3.0	
>15	956	97.5	523	97.0	
**Lymphatic vessel infiltration**					0.326
Negative	502	51.2	290	53.8	
Positive	479	48.8	249	46.2	
**Postoperative complications**					**0.001**
No	823	83.9	413	76.6	
Yes	158	16.1	126	23.4	
**Clavien–Dindo grade**					**<0.001**
None	823	83.9	413	76.6	
I–II	133	13.6	76	14.1	
III–IV	25	2.5	50	9.3	
**Chemotherapy**					
AC group	981	100	0	0.0	
UAC group	0	0	539	100.0	
Delay (>60 days)	0	0	62	11.5	
Omission	0	0	477	88.5	
**Pathological differentiation degree**					0.342
Differentiated	753	76.8	402	74.6	
Undifferentiated	228	23.2	137	25.4	
**Follow-up, month**					
Median	43	37			
Range	3–86	3–91			

*AC, Acceptable chemotherapy; UAC, unacceptable delay or missing chemotherapy; pT, pathological tumor; pN, pathological node*.

*Bold values indicates P < 0.05, statistically significant*.

### Effect of Unacceptable Chemotherapy Initiation on Prognosis

**Figure 1:**
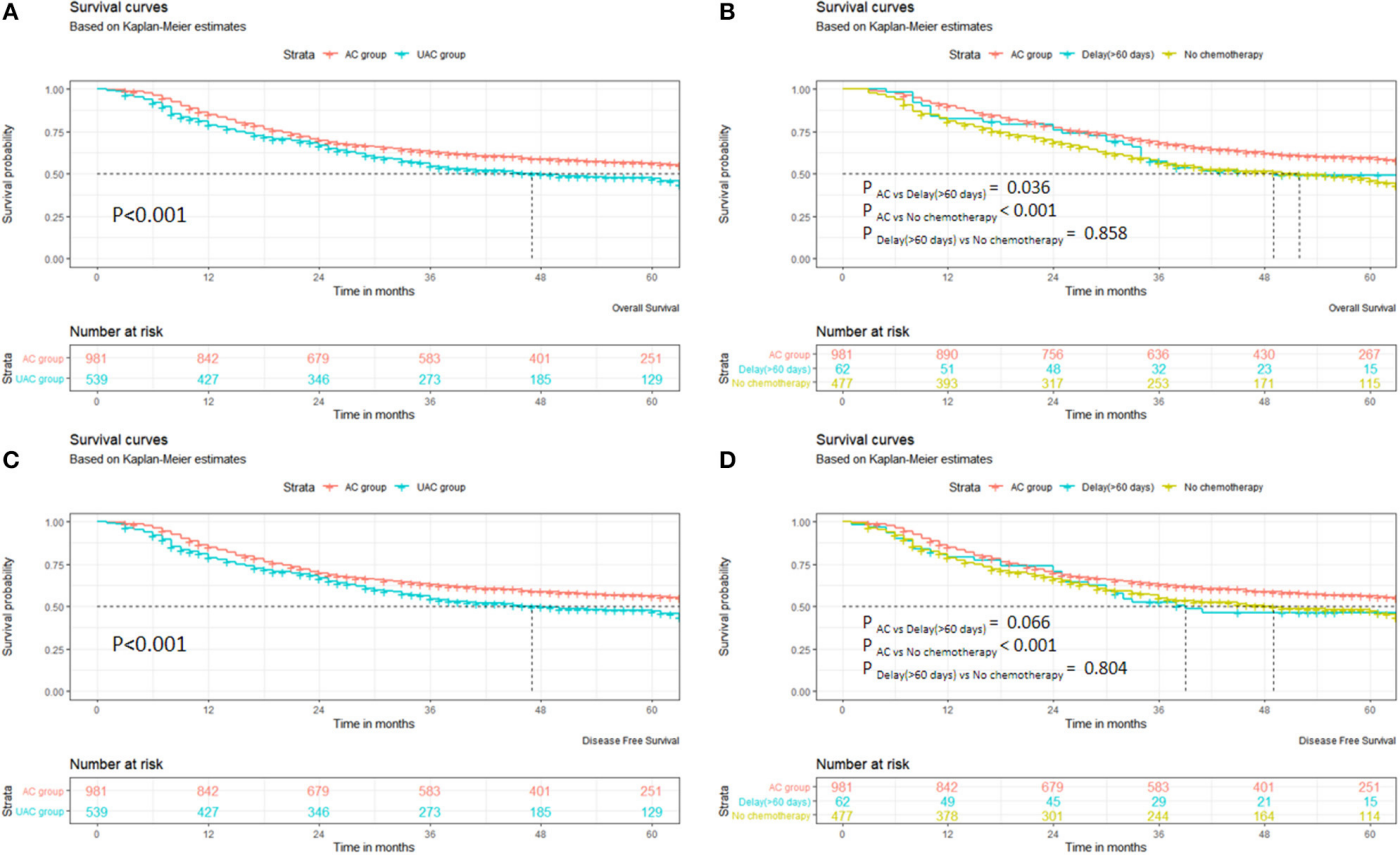
Survival of patients in AC and UAC groups. **(A)** OS of AC group vs. UAC group. **(B)** OS among AC group, delay (>60 days), and no chemotherapy. **(C)** DFS of AC group vs. UAC group. **(D)** DFS among AC group, delay (>60 days), and no chemotherapy.

Supplementary Figure 3 shows the OS of the 1,520 patients, with a median survival time of 41 months (range 3–91 months). Figure 1 indicates that the OS and DFS of the UAC group patients are significantly inferior to those in the AC group (*p* < 0.001). In the UAC group, the OS and DFS of the patients with chemotherapy delayed until more than 60 days were close to those without chemotherapy (*p* > 0.05). Further analysis shows that the AC group had significantly better OS than the UAC group (*p* < 0.05) in both men/women, with or without complications, or stage II/III (Supplementary Figure 4).

The stratified analysis by stage II/III shows that the OS in the AC group in the IIA–IIIC patients was significantly better than that in the UAC group (*P* < 0.05). When the DFS was compared, the AC group was significantly superior to the UAC group in both males and females, with or without complications, or stage II/III patients (*P* < 0.05) (Supplementary Figure 5). A further stratified analysis by stage II/III demonstrates that the DFS in the AC Group exhibited a greater trend than that in the UAC group in the IIb, IIIA, and IIIC patients; however, the differences were not significant (*p* > 0.05). In contrast, in the AC group in the IIA and IIIB patients, the DFS was significantly better than that in the UAC group (*p* < 0.05). Supplementary Figure 6 shows that extended survival after recurrence in the AC group was superior to that in the UAC group (*P* = 0.014).

### Analysis of Risk Factors for Patient Survival

Table 2 shows the results of the Cox univariate and multivariate analyses for OS. The univariate analysis indicates that age, BMI, medical insurance type, ASA score, comorbidities, tumor site, pTNM staging, tumor size, lymphatic vessel infiltration, pathological differentiation degree, number of examined lymph nodes, Clavien–Dindo grade, and UAC were associated with OS (all *p* < 0.05). The multivariate analysis indicates that age, BMI, ASA score, comorbidities, pTNM staging, number of examined lymph nodes, Clavien–Dindo grade, and UAC were independent predictors of OS (*P* < 0.05). Supplementary Table 1 demonstrates that age, pTNM staging, tumor size, and number of harvested lymph nodes were independent predictors of DFS; however, UAC was not an independent risk factor (*p* < 0.05).

**Table 2:** Univariate and multivariate Cox regression models for overall survival.

**Variable**		**Univariate model**				**Full multivariate model**				**Reduced multivariate model**			
		**HR**	**95% CI**		***P***	**HR**	**95% CI**		***P***	**HR**	**95% CI**		***P***
Patients' risk	**Sex**				0.565								
	Female	Ref											
	Male	1.05	0.89	1.25	0.565								
	**Age, year**				**0.002**				**0.003**				**0.003**
	<65	Ref				Ref				Ref			
	≥65	1.27	1.09	1.48	**0.002**	1.29	1.09	1.53	**0.003**	1.29	1.09	1.52	**0.003**
	**BMI**				**<0.001**				**0.003**				**0.004**
	<18.5	Ref				Ref				Ref			
	18.5–24.9	0.60	0.49	0.75	**<0.001**	0.74	0.59	0.91	**0.006**	0.74	0.59	0.92	**0.007**
	≥25.0	0.50	0.37	0.66	**<0.001**	0.60	0.45	0.81	**0.001**	0.61	0.45	0.82	**0.001**
	**Medical insurance type**				**0.046**								
	Self-paid	Ref											
	Rural insurance	0.71	0.56	0.91	**0.006**								
	Urban insurance	0.73	0.57	0.94	**0.015**								
	Others	0.95	0.35	2.60	0.923								
	**Occupation**				0.872								
	No	Ref											
	Yes	1.03	0.74	1.43	0.872								
	**Income**				0.218								
	Very low	Ref											
	Low	0.86	0.71	1.04	0.121								
	General	0.96	0.76	1.22	0.745								
	High	0.66	0.39	1.12	0.124								
	**Social status**				0.426								
	Very low	Ref											
	Low	0.99	0.52	1.87	0.978								
	General	0.88	0.47	1.65	0.692								
	High	0.73	0.37	1.46	0.375								
	Very high	0.91	0.40	2.11	0.832								
	**Residential address**				0.745								
	Village	Ref											
	City	0.98	0.84	1.14	0.745								
	**Marital status**				0.515								
	No	Ref											
	Yes	0.80	0.42	1.55	0.515								
	**Procreation status**				0.209								
	No	Ref											
	Yes	0.68	0.38	1.24	0.209								
	**Smoking and drinking consumption**				0.312								
	No	Ref											
	Smoking	0.88	0.72	1.06	0.167								
	Drinking	1.26	0.83	1.90	0.273								
	Both	0.95	0.75	1.21	0.683								
	**ASA score**				**0.043**				**0.002**				**0.002**
	I	Ref				Ref				Ref			
	II	1.10	0.94	1.29	0.252	1.24	0.98	1.58	0.072	1.25	0.98	1.59	0.068
	III–IV	1.51	1.08	2.11	**0.016**	2.09	1.39	3.15	**<0.001**	2.09	1.38	3.14	**<0.001**
	**Abdominal surgery history**				0.675								
	No	Ref											
	Yes	1.05	0.84	1.31	0.675								
	**Intra-abdominal surgery history**				0.615								
	No	Ref											
	Yes	1.07	0.81	1.41	0.615								
	**Comorbidity**				**<0.001**				**0.043**				**0.040**
	No	Ref				Ref				Ref			
	Yes	1.47	1.23	1.76	**<0.001**	1.32	1.01	1.72	**0.043**	0.76	0.58	0.99	**0.040**
Tumor's risk	**Tumor site**				**<0.001**				0.111				0.098
	Lower	Ref				Ref				Ref			
	Middle	1.27	1.03	1.57	0.023	1.18	0.96	1.47	0.122	1.18	0.95	1.46	0.126
	Upper	1.12	0.92	1.36	0.280	1.06	0.86	1.30	0.589	1.06	0.86	1.29	0.591
	Overlapping lesion of stomach	1.91	1.53	2.38	**<0.001**	1.32	1.04	1.68	**0.024**	1.33	1.05	1.69	**0.020**
	**pTNM stage**				**<0.001**				**<0.001**				**<0.001**
	IIA	Ref				Ref				Ref			
	IIB	1.24	0.86	1.80	0.248	1.21	0.83	1.76	0.320	1.23	0.84	1.78	0.288
	IIIA	1.72	1.21	2.43	**0.002**	1.67	1.17	2.37	**0.005**	1.69	1.19	2.41	**0.004**
	IIIB	3.08	2.27	4.19	**<0.001**	2.81	2.04	3.86	**<0.001**	2.91	2.13	3.99	**<0.001**
	IIIC	5.41	4.02	7.28	**<0.001**	4.42	3.22	6.08	**<0.001**	4.64	3.39	6.34	**<0.001**
	**Tumor size, mm**				**<0.001**				**0.013**				**0.012**
	<20	Ref				Ref				Ref			
	20–50	1.37	0.78	2.38	0.272	1.00	0.57	1.77	0.997	1.01	0.57	1.78	0.975
	>50	2.60	1.50	4.53	**0.001**	1.29	0.73	2.28	0.385	1.30	0.73	2.29	0.370
	**Lymphatic vessel infiltration**				**<0.001**				0.231				
	Negative	Ref				Ref							
	Positive	1.32	1.14	1.54	**<0.001**	1.10	0.94	1.29	0.231				
	**Pathological differentiation degree**				**0.001**				0.172				
	Differentiated	Ref				Ref							
	Undifferentiated	0.72	0.60	0.87	**0.001**	0.87	0.71	1.06	0.172				
Treatment risk	**Examined LNs, no**.				**<0.001**				**<0.001**				**<0.001**
	>15	Ref				Ref				Ref			
	≤ 15	2.15	1.47	3.14	**<0.001**	2.82	1.90	4.19	**<0.001**	2.72	1.84	4.03	**<0.001**
	**Clavien–Dindo grade**				**<0.001**				**0.037**				**0.033**
	None	Ref				Ref				Ref			
	I–II	1.41	1.14	1.74	**0.001**	1.29	1.04	1.60	**0.023**	1.29	1.04	1.60	**0.022**
	III–IV	1.64	1.21	2.23	**0.002**	1.27	0.93	1.75	0.139	1.28	0.93	1.77	0.124
	**Chemotherapy**				**<0.001**				**0.003**				**0.004**
	AC group	Ref				Ref				Ref			
	UAC group	1.48	1.27	1.73	**<0.001**	1.29	1.09	1.52	**0.003**	1.28	1.08	1.51	**0.004**

*AC, Acceptable chemotherapy; UAC, unacceptable delay or missing chemotherapy; LN, lymph node; HR, hazard ratio*.

*Bold values indicates P < 0.05, statistically significant*.

### Analysis of Risk Factors of Unacceptable Chemotherapy Initiation

**Figure 2:**
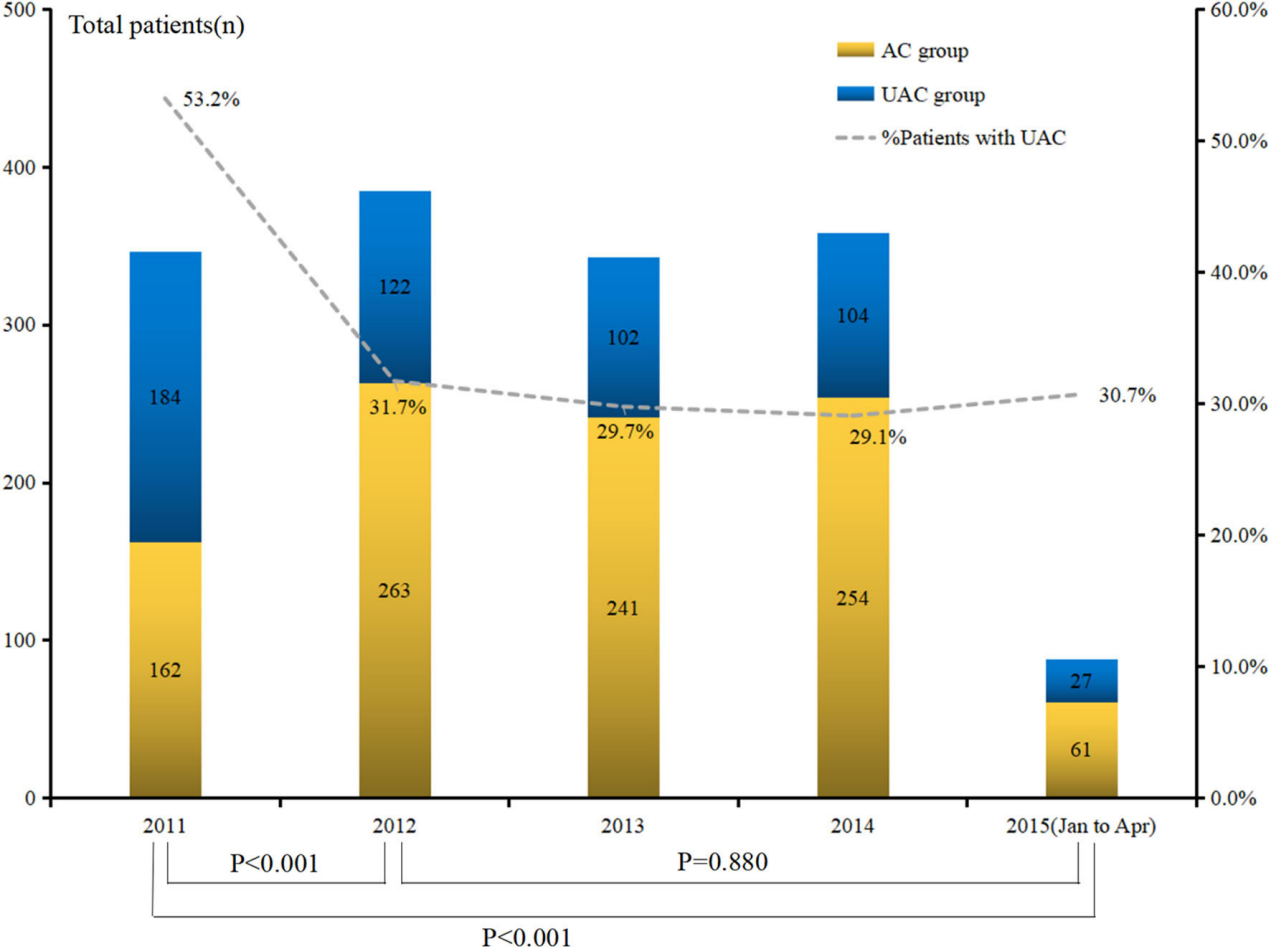
Trend of unacceptable chemotherapy in pathological stage II/III gastric cancer patients between 2011 and April 2015.

The analysis of the UAC status from 2011 to April 2015 showed that the rates in 2011, 2012, 2013, 2014, and 2015 were 53.2, 31.7, 29.7, 29.1, and 30.7%, respectively (Figure 2). The difference reached statistical significance (*P* < 0.001). Further stratified analysis showed a significant improvement in UAC in 2012 compared with that in 2011 (*p* < 0.001), whereas there was no significant improvement from 2012 to April 2015 (*p* = 0.880); moreover, it increased from 29.1% in 2014 to 30.7% in 2015. Table 3 presents the results of the logistic univariate and multivariate analyses of risk factors associated with UAC. The univariate analysis shows that sex, age, type of medical insurance, income, social status, residential address, ASA score, history of intra-abdominal surgery, Charlson score, and Clavien–Dindo grade were related to UAC (all *p* < 0.05). The reduced multivariate model analysis shows that female sex, old age, self-paid status, very low social status, high ASA score, intra-abdominal surgery history, and serious postoperative complications (Clavien–Dindo III–IV) were independent risk factors for UAC (*p* < 0.05).

**Table 3:** Univariate and multivariate logistic models for the risks of unacceptable chemotherapy.

**Variable**		**Univariate model**				**Full Multivariate model**				**Reduced multivariate model**			
		**OR**	**95% CI**		***P***	**OR**	**95% CI**		***P***	**OR**	**95% CI**		***P***
Patients' risk	**Sex**				**0.030**				**0.011**				**0.015**
	Female	Ref				Ref				Ref			
	Male	0.77	0.61	0.98	**0.030**	0.72	0.55	0.93	**0.011**	0.73	0.56	0.94	**0.015**
	**Age, year**				**<0.001**				**<0.001**				**<0.001**
	<65	Ref				Ref				Ref			
	≥65	2.80	2.25	3.47	**<0.001**	2.47	1.92	3.19	**<0.001**	2.45	1.91	3.14	**<0.001**
	**BMI**				0.470								
	<18.5	Ref											
	18.5–24.9	0.83	0.60	1.14	0.246								
	≥25.0	0.80	0.53	1.20	0.279								
	**Medical insurance type**				**<0.001**				**<0.001**				**<0.001**
	Self-paid	Ref				Ref				Ref			
	Rural insurance	0.38	0.27	0.54	**<0.001**	0.39	0.27	0.57	**<0.001**	0.39	0.26	0.57	**<0.001**
	Urban insurance	0.47	0.33	0.68	**<0.001**	0.38	0.25	0.57	**<0.001**	0.36	0.24	0.55	**<0.001**
	Others	0.42	0.10	1.72	0.226	0.18	0.04	0.93	**0.040**	0.19	0.04	0.92	**0.040**
	**Occupation**				0.534								
	No	Ref											
	Yes	1.17	0.72	1.90	0.534								
	**Income**				**0.004**				0.186				
	Very low	Ref				Ref							
	Low	0.64	0.49	0.83	**0.001**	1.40	0.94	2.08	0.094				
	General	0.73	0.52	1.01	0.060	1.17	0.69	1.98	0.563				
	High	0.43	0.20	0.91	**0.028**	0.63	0.19	2.09	0.447				
	**Social status**				**<0.001**				**0.001**				**0.001**
	Very low	Ref				Ref				Ref			
	Low	0.28	0.10	0.80	**0.017**	0.25	0.08	0.73	**0.012**	0.26	0.09	0.78	**0.016**
	General	0.14	0.05	0.40	**<0.001**	0.14	0.05	0.43	**0.001**	0.18	0.06	0.52	**0.002**
	High	0.23	0.08	0.67	**0.007**	0.19	0.06	0.67	**0.009**	0.19	0.06	0.62	**0.005**
	Very high	0.12	0.03	0.47	**0.002**	0.14	0.02	0.84	**0.032**	0.08	0.02	0.36	**0.001**
	**Residential address**				**0.011**				0.063				0.072
	Village	Ref				Ref				Ref			
	City	1.32	1.07	1.63	**0.011**	1.34	0.98	1.82	0.063	1.31	0.98	1.76	0.072
	**Marital status**				0.544								
	No	Ref											
	Yes	0.75	0.30	1.88	0.544								
	**Procreation status**				0.799								
	No	Ref											
	Yes	0.89	0.37	2.16	0.799								
	**Smoking and drinking consumption**				0.212								
	No	Ref											
	Smoking	0.84	0.65	1.09	0.198								
	Drinking	0.82	0.44	1.54	0.534								
	Both	0.72	0.52	1.02	0.062								
	**ASA score**				**<0.001**				0.129				**0.047**
	I	Ref				Ref				Ref			
	II	1.56	1.25	1.95	**<0.001**	1.24	0.88	1.75	0.212	1.26	0.99	1.60	0.063
	III–IV	2.46	1.48	4.09	**<0.001**	1.96	1.01	3.81	**0.047**	1.74	1.01	3.02	**0.047**
	**Abdominal surgery history**				**<0.001**				**0.002**				**0.003**
	No	Ref				Ref				Ref			
	Yes	1.75	1.30	2.38	**<0.001**	1.65	1.18	2.29	**0.002**	1.65	1.19	2.30	**0.003**
	**Intra-abdominal surgery history**				0.501								
	No	Ref											
	Yes	1.15	0.77	1.70	0.501								
	**Charlson score**				**<0.001**				0.344				
	0	Ref				Ref							
	1–2	1.68	1.33	2.11	**<0.001**	0.95	0.65	1.40	0.798				
	3–5	0.99	0.40	2.44	0.978	0.43	0.14	1.32	0.140				
Tumor's risk	**Tumor site**				0.684								
	Lower	Ref											
	Middle	0.89	0.67	1.18	0.415								
	Upper	1.05	0.81	1.36	0.731								
	Overlapping lesion of stomach	1.07	0.77	1.49	0.688								
	**pTNM stage**				0.224								
	IIA	Ref											
	IIB	0.86	0.59	1.24	0.413								
	IIIA	0.72	0.49	1.05	0.084								
	IIIB	1.05	0.75	1.46	0.791								
	IIIC	0.99	0.71	1.37	0.929								
	**Tumor size, mm**				0.105								
	<20	Ref											
	20–50	0.90	0.49	1.66	0.739								
	>50	1.14	0.62	2.10	0.680								
	**Lymphatic vessel infiltration**				0.326								
	Negative	Ref											
	Positive	0.90	0.73	1.11	0.326								
	**Pathological differentiation degree**				0.342								
	Differentiated	Ref											
	Undifferentiated	1.13	0.88	1.44	0.342								
Treatment risk	**Examined LNs, no**.				0.629								
	>15	Ref											
	≤15	1.17	0.62	2.21	0.629								
	**Clavien–Dindo grade**				**<0.001**				**<0.001**				**<0.001**
	None	Ref				Ref				Ref			
	I–II	1.14	0.84	1.55	0.405	1.13	0.82	1.57	0.447	1.14	0.83	1.58	0.427
	III–IV	3.99	2.43	6.53	**<0.001**	3.83	2.25	6.53	**<0.001**	3.73	2.20	6.33	**<0.001**

*AC, Acceptable chemotherapy; UAC, unacceptable delay or missing chemotherapy; LN, lymph node; OR, odds ratio*.

*Bold values indicates P < 0.05, statistically significant*.

### Change of Risk Factors for Unacceptable Chemotherapy Initiation Over Time

The radar chart shows that the risk factors of UAC changed with time (Supplementary Figure 7). In general, the number of patients with self-funded and an extremely low social status significantly decreased from 2011 to April 2015 (all *p* < 0.05). The proportion of self-funded patients showed a downward trend year by year from 2011 to April 2015, whereas the proportion of extremely low social status patients gradually decreased (0–3.2%) from 2011 to 2014 and then increased again (4.5%) in 2015 (Supplementary Table 2). Between 2011 and April 2015, there was no significant change in the proportion of female patients, aged patients, patients undergoing intra-abdominal surgery, and high ASA patients (all *p* > 0.05). At the same time, the proportion of patients with severe postoperative complications was not significantly improved (*p* = 0.549).

## Discussion

It has been confirmed in animal models that the angiogenesis of the micrometastatic foci will be significantly faster after resection of the primary cancer (19–21); thus, the treatment of advanced cancer cannot rely solely on surgical treatment (22–25). Therefore, although surgery is a key part of the comprehensive treatment for locally advanced GC, timely adjuvant chemotherapy plays an important role. Our results show that the patients with a chemotherapy delay (delayed more than 60 days) or without chemotherapy have significantly worse overall survival and DFS than those with timely chemotherapy. The patients with delayed or omitted chemotherapy did not exhibit significant differences, and the chemotherapy delay patients' sample size was limited. Thus, we combined them in the subsequent analysis as UAC. Our data show that UAC is a risk factor for OS independent of tumor staging, age, and other factors for stage II/III GC patients. The reason may be that a chemotherapy delay can affect the early inhibition of cytotoxic drugs on angiogenesis in micrometastasis, and it is easy to induce primary drug resistance (21, 26). Several reasons for this are possible. Firstly, UAC is not an independent risk factor for the DFS of all patients with stage II/III GC, and the reason may be that extended survival after recurrence in the AC group was superior to that in the UAC group. Previous studies also showed that extended survival after recurrence may influence the effect of treatment on prognosis and reduce the association between treatment effects on DFS and OS, where delay/omission of chemotherapy was a significant factor for OS but not DFS (27). Some patients may have a recurrence after surgery and mostly could be seen on physical examination or follow-up examination. However, due to the limited detection methods at present, some recurrence may be missed, especially in patients with concealed relapses. Therefore, some patients could have undiagnosed tumor recurrences, leading to loss of data. These factors may affect the results. These could be the reason why delay/omission of chemotherapy was a significant factor for OS but not for DFS.

As an independent risk factor for OS, clinicians should avoid the occurrence of UAC in patients. The rate of UAC in recent years was significantly reduced from 2011 to 2012; however, the rate did not significantly decrease from 2012 to 2015 and increased from 29.1% in 2014 to 30.7% in 2015. Therefore, it is of importance to identify the high-risk factors of UAC and investigate their changes overtime to facilitate relevant departments to use appropriate measures to improve this situation. At the same time, we believe that the causes of UAC are not only related to clinical treatment factors, such as surgery, postoperative complications, and tumor factors, and individual factors, such as socioeconomic variables, may also play important roles. Therefore, we expanded the included variables, such as the medical insurance type, according to the characteristics of the regional socioeconomic factors, and divided the included factors into three categories, including individual, treatment, and tumor, for logistic analysis. The results showed that female, elderly, self-paid patients and patients with a low social status, high ASA score, preoperative abdominal operation history, and severe postoperative complications exhibited high-risk factors of UAC, whereas tumor factors, such as tumor size and staging, did not affect postoperative chemotherapy time. Further analysis indicated the risk factors that changed over time in the intervening factors, with the increase in time and the surgical experience accumulated, whereas serious postoperative complications did not significantly improve. Severe postoperative complications often require longer recovery times and may affect patients' confidence in themselves and their doctors' treatment (28), thus delaying their first time of receiving chemotherapy. Moreover, low-status groups typically suffer from substantial life pressures; thus, they often delay or give up chemotherapy due to personal or family factors after surgery. Therefore, although through the unremitting efforts of the government, the proportion of self-financed patients decreases year by year (which can improve the patient's willingness and timeliness of chemotherapy), the rate of UAC has not significantly improved in recent years.

We believe that to improve the OS of GC, the relevant departments or personnel should take corresponding measures in response to the various factors that cause UAC. For clinical workers, preoperative evaluation, intraoperative quality control, and enhanced postoperative management should be performed to reduce postoperative complications, particularly severe postoperative complications. At the same time, postoperative care and education should receive attention in high-risk groups, such as individuals with a low social status, to inform them of the importance of timely postoperative chemotherapy. The government should continue to improve the medical insurance policy to continue to reduce the proportion of self-funded patients.

This paper incorporates multiple factors to explore high-risk groups to provide a reference for relevant departments and personnel to use corresponding measures. However, our research has unavoidable shortcomings: First, as a retrospective study, it is difficult to exclude the effects of confounding factors on the results, that is, the patient's personal preferences may have an impact on the UAC. Second, there are significant differences in multiple factors between the UAC group and the AC group. This imbalance may have an impact on the subsequent prognosis analysis. Some patients, such as those with a very low social status, are limited in number at the time of stratification, which may affect the results. Third, the risk factors for UAC in different countries may vary due to different national conditions, such as health care policies and income conditions (29, 30). Nevertheless, the results of this retrospective study are important. We also look forward to international, multicenter, retrospective studies in the near future to explore the risk factors and their differences in each country, thus providing strategies for improving the overall prognosis of patients with GC worldwide.

## Data Availability Statement

The raw data supporting the conclusions of this article will be made available by the authors, without undue reservation, to any qualified researcher.

## Ethics Statement

The studies involving human participants were reviewed and approved by the Research Ethics Committee at the Fujian Medical University Union Hospital (the reference number is 2019KY063). Written informed consent for participation was not required for this study in accordance with the national legislation and the institutional requirements.

## Author Contributions

Q-YC, Z-YL, QZ, and C-MH: conception and design. C-MH: provision of study materials or patients. Q-YC, Z-YL, QZ, C-MH, PL, H-LZ, J-XL, J-WX, J-BW, JL, L-LC, J-LL, and R-HT: collection and/or assembly of data. C-MH, Q-YC, Z-YL, QZ, and Z-NH: data analysis and interpretation. C-MH, PL, Q-YC, QZ, ML, and H–LZ: manuscript writing. All authors read and approved the final manuscript.

## Conflict of Interest

The authors declare that the research was conducted in the absence of any commercial or financial relationships that could be construed as a potential conflict of interest. The handling editor declared a past co-authorship with one of the authors C-HZ.

## Abbreviations

UAC group: Unacceptable chemotherapy initiation
AC group: Acceptable chemotherapy initiation
GC: Gastric cancer
FMUUH: Fujian Medical University Union Hospital
CT: Computed tomography
US: Ultrasonography
PET-CT: Positron emission tomography–computed tomography
UICC: International Union Against Cancer
OS: Overall survival
DFS: Disease-free survival.
