# Supplementary material for: Clinical Impact of Delayed Initiation of Adjuvant Chemotherapy Among Patients With Stage II/III Gastric Cancer: Can We Do Better?
Source: Front Oncol. 2020 Jul 31;10:1149. doi: 10.3389/fonc.2020.01149 (PMC7412732; doi:10.3389/fonc.2020.01149)
Supplement: Supplementary file 1 [file Data_Sheet_2.docx]

**Figure legends:**


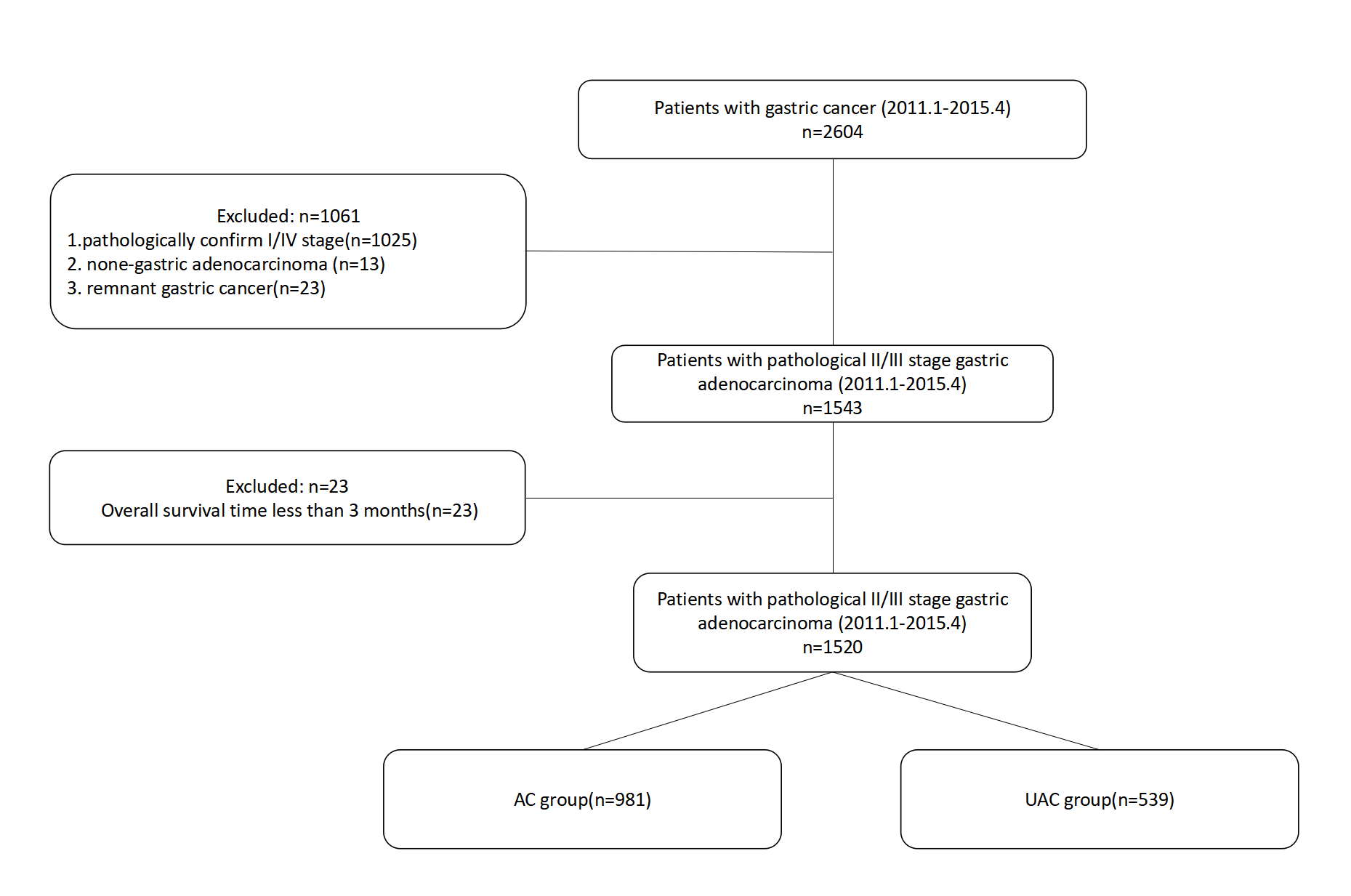


Supplementary Fig.1 Study cohort


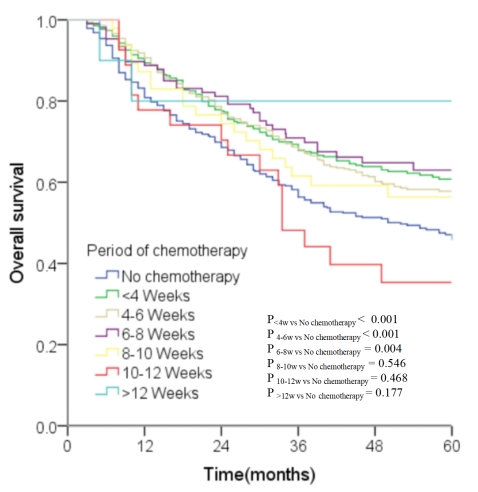


Supplementary Fig. 2 The relationship between overall survival and time to first chemotherapy initiation.


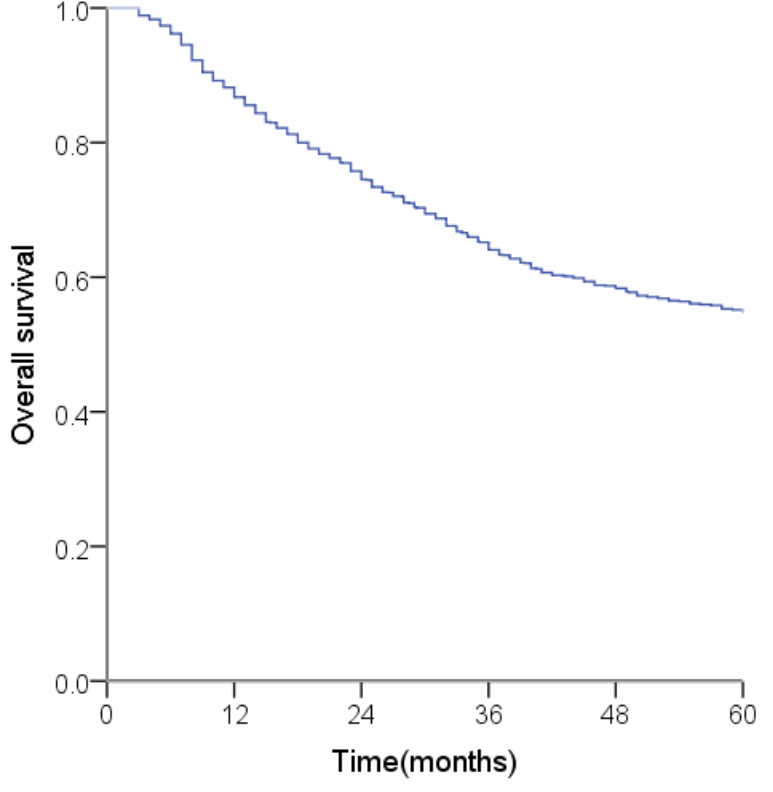


Supplementary Fig. 3 Overall survival in all patients


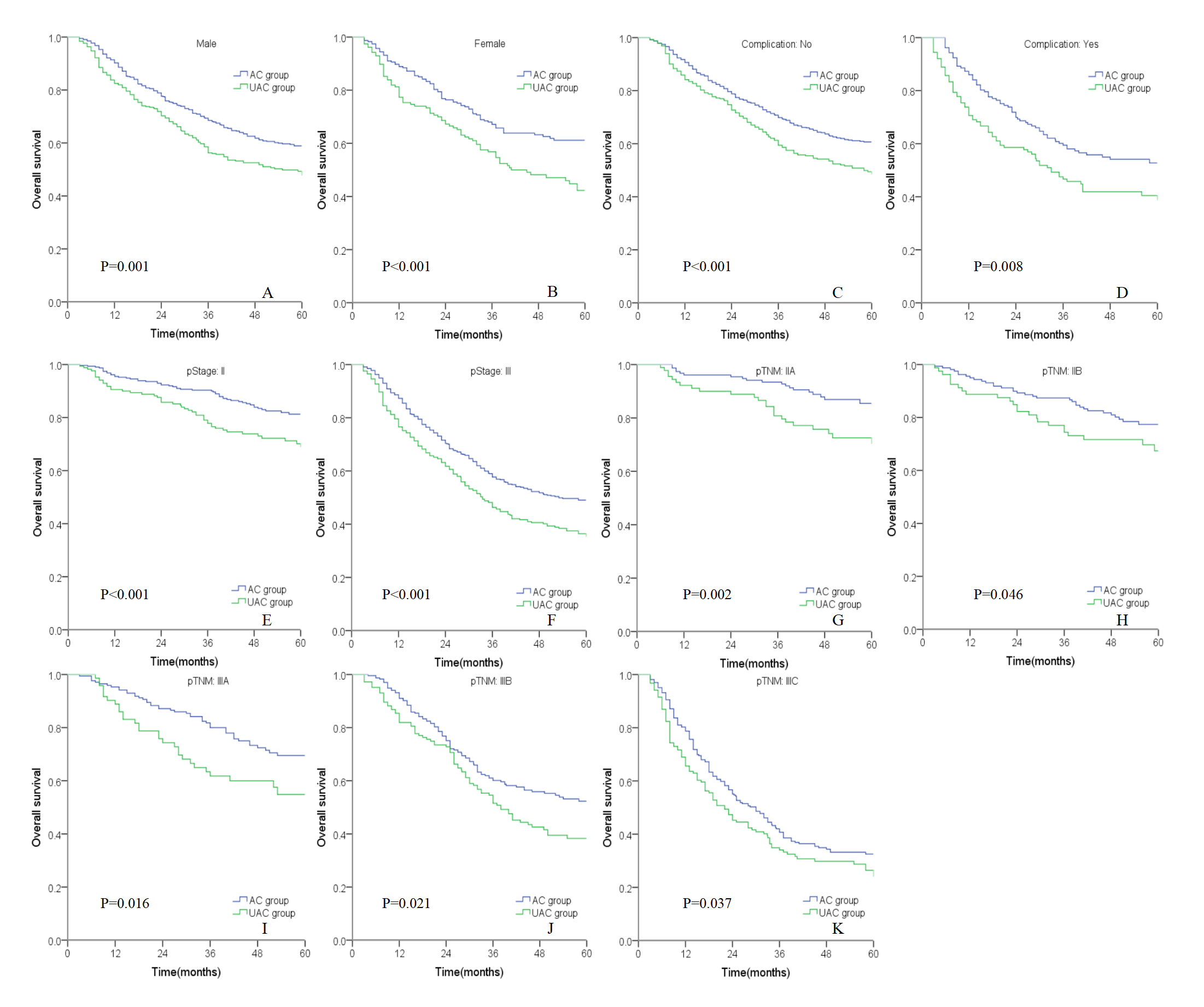


Supplementary Fig. 4 Stratified analysis of OS between AC and UAC groups. (A: Male, B: Female; C: IIA, D: IIB, E: IIIA; F: IIIB; G: IIIC; H: No complication; I: Complication; J: Clavien-Dindo None; K: Clavien-Dindo I-II; L: Clavien-Dindo III-IV).


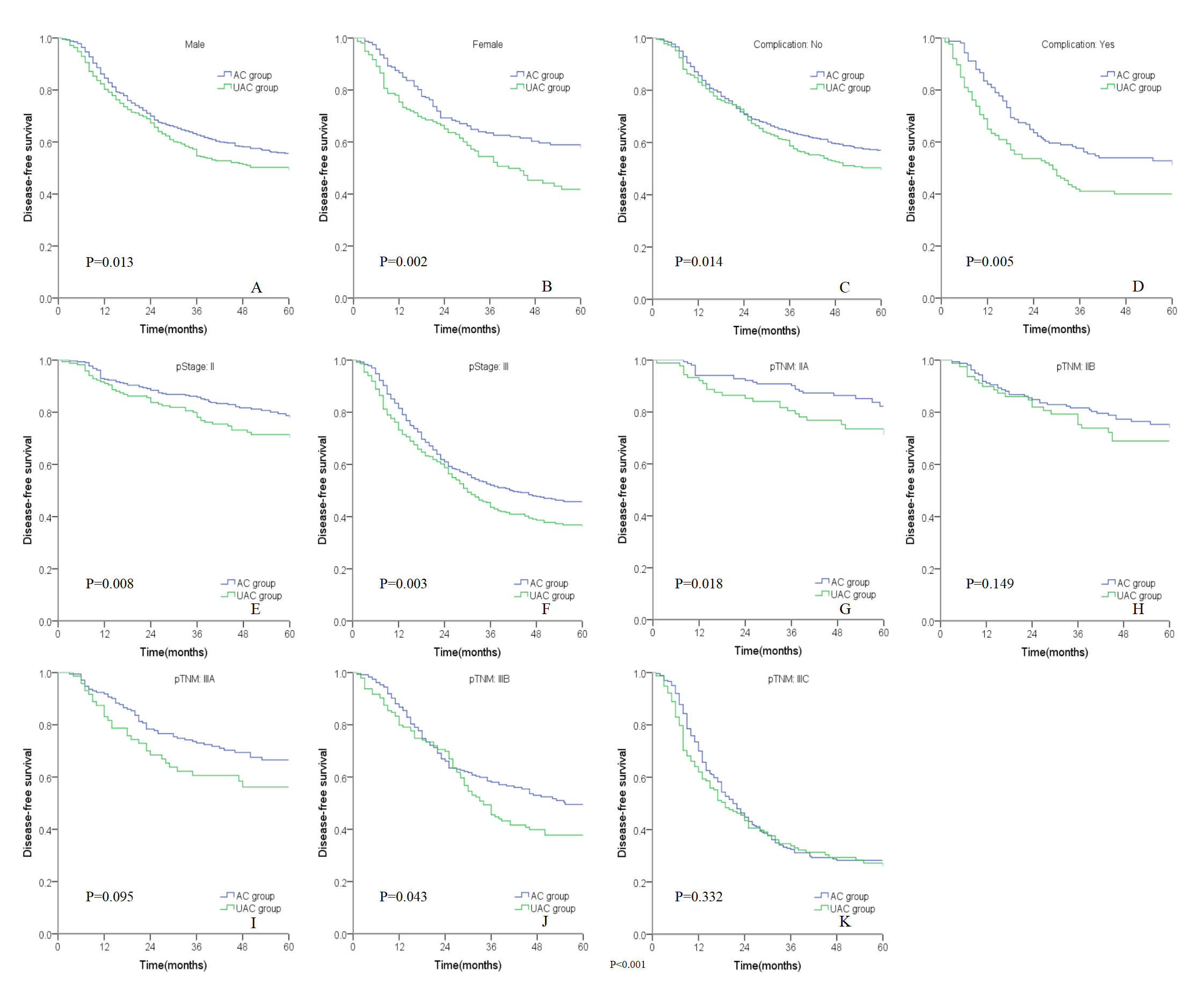


Supplementary Fig. 5 Stratified analysis of DFS between AC and UAC groups. (A: Male, B: Female; C: IIA, D: IIB, E: IIIA; F: IIIB; G: IIIC; H: No complication; I: Complication; J: Clavien-Dindo None; K: Clavien-Dindo I-II; L: Clavien-Dindo III-IV).


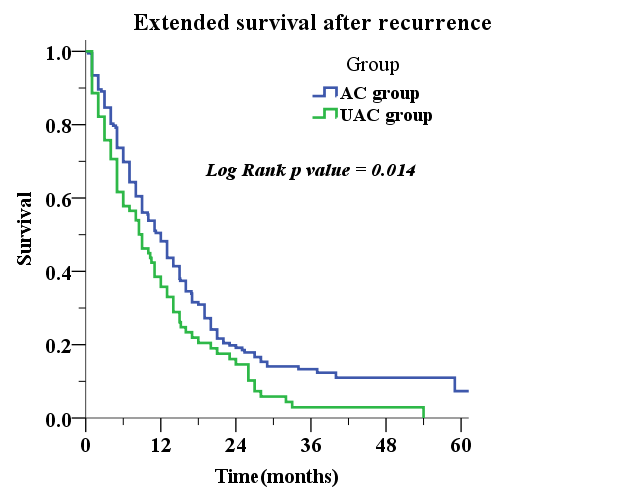


Supplementary Fig. 6 Survival analysis of extended survival after recurrence in gastric cancer patients.


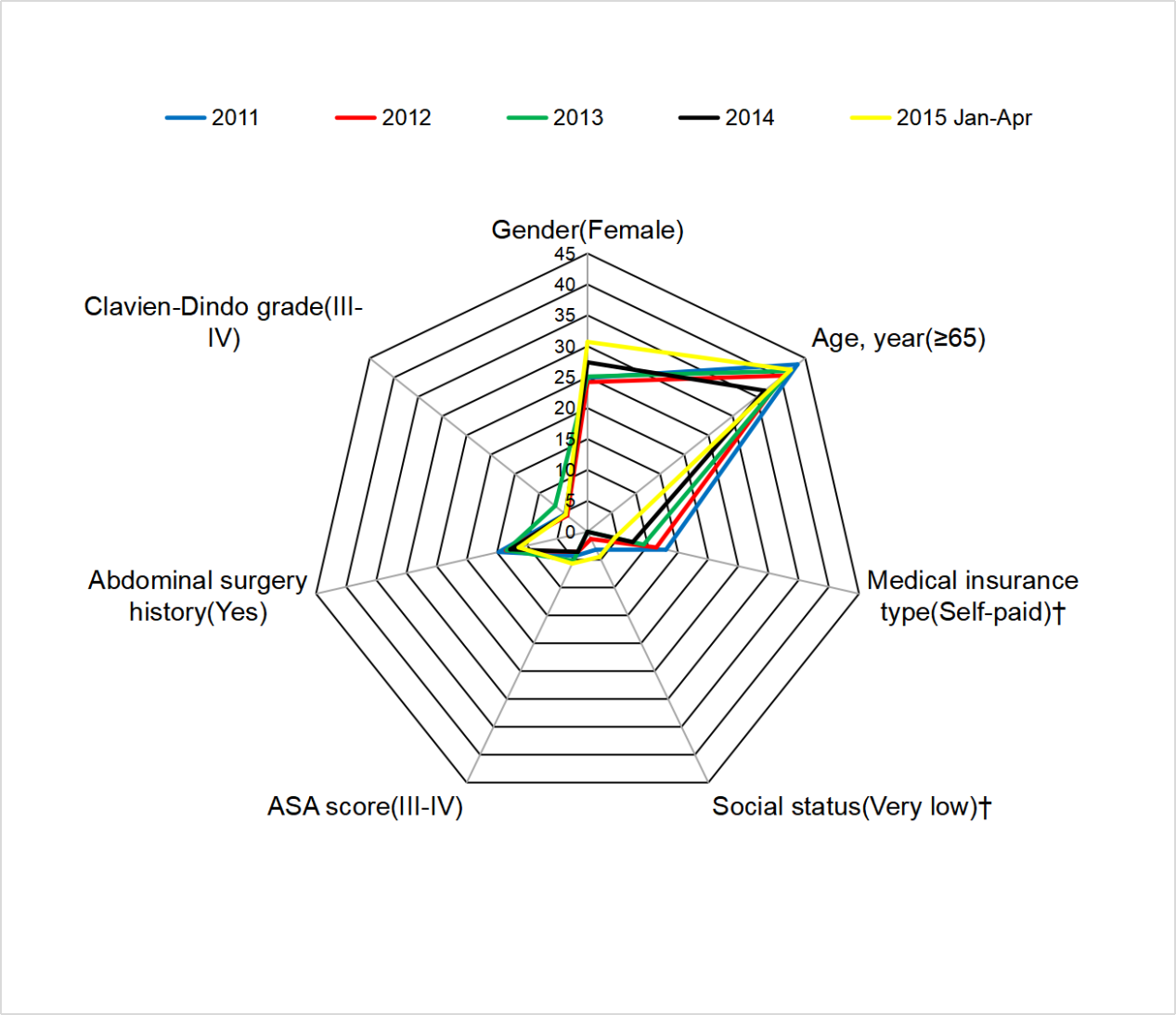


Supplementary Fig. 7 Analysis of the risk factors of unacceptable chemotherapy from 2011 to April 2015, † P value <0.05.
