# Supplementary material for: Clinical Impact of Delayed Initiation of Adjuvant Chemotherapy Among Patients With Stage II/III Gastric Cancer: Can We Do Better?
Source: Front Oncol. 2020 Jul 31;10:1149. doi: 10.3389/fonc.2020.01149 (PMC7412732; doi:10.3389/fonc.2020.01149)
Supplement: Supplementary file 2 [file Data_Sheet_1.doc]

Supplementary Table 1. Univariate and multivariate cox regression models for disease free survival

| Variable | | Univariate Model | | | | Full Multivariate Model | | | | Reduced Multivariate Model | | | |
| --- | --- | --- | --- | --- | --- | --- | --- | --- | --- | --- | --- | --- | --- |
|  |  | HR | 95%CI | | P | HR | 95%CI | | P | HR | 95%CI | | P |
| Patients' risk | **Gender** |  |  |  | 0.737 |  |  |  |  |  |  |  |  |
|  | Female | Ref |  |  |  |  |  |  |  |  |  |  |  |
|  | Male | 1.03 | 0.87 | 1.22 | 0.737 |  |  |  |  |  |  |  |  |
|  | **Age,year** |  |  |  | **0.025** |  |  |  | **0.020** |  |  |  | **0.022** |
|  | <65 | Ref |  |  |  | Ref |  |  |  | Ref |  |  |  |
|  | ≥65 | 1.19 | 1.02 | 1.38 | **0.025** | 1.21 | 1.03 | 1.41 | **0.020** | 1.20 | 1.03 | 1.40 | **0.022** |
|  | **BMI** |  |  |  | **<0.001** |  |  |  | 0.086 |  |  |  |  |
|  | <18.5 | Ref |  |  |  | Ref |  |  |  |  |  |  |  |
|  | 18.5-24.9 | 0.69 | 0.56 | 0.85 | **0.001** | 0.85 | 0.68 | 1.05 | 0.135 |  |  |  |  |
|  | ≥25.0 | 0.58 | 0.43 | 0.77 | **<0.001** | 0.72 | 0.54 | 0.96 | **0.027** |  |  |  |  |
|  | **Medical insurance type** |  |  |  | 0.186 |  |  |  |  |  |  |  |  |
|  | Self-paid | Ref |  |  |  |  |  |  |  |  |  |  |  |
|  | Rural-insurance | 0.77 | 0.60 | 0.98 | **0.033** |  |  |  |  |  |  |  |  |
|  | Urban-insurance | 0.84 | 0.65 | 1.08 | 0.168 |  |  |  |  |  |  |  |  |
|  | Others | 0.89 | 0.32 | 2.42 | 0.812 |  |  |  |  |  |  |  |  |
|  | **Occupation** |  |  |  | 0.424 |  |  |  |  |  |  |  |  |
|  | No | Ref |  |  |  |  |  |  |  |  |  |  |  |
|  | Yes | 1.14 | 0.83 | 1.57 | 0.424 |  |  |  |  |  |  |  |  |
|  | **Income** |  |  |  | 0.623 |  |  |  |  |  |  |  |  |
|  | Very low | Ref |  |  |  |  |  |  |  |  |  |  |  |
|  | Low | 0.97 | 0.80 | 1.18 | 0.782 |  |  |  |  |  |  |  |  |
|  | General | 1.11 | 0.88 | 1.40 | 0.386 |  |  |  |  |  |  |  |  |
|  | High | 1.00 | 0.62 | 1.62 | 0.987 |  |  |  |  |  |  |  |  |
|  | **Social status** |  |  |  | 0.791 |  |  |  |  |  |  |  |  |
|  | Very low | Ref |  |  |  |  |  |  |  |  |  |  |  |
|  | Low | 0.98 | 0.52 | 1.85 | 0.945 |  |  |  |  |  |  |  |  |
|  | General | 0.93 | 0.50 | 1.74 | 0.824 |  |  |  |  |  |  |  |  |
|  | High | 0.81 | 0.41 | 1.61 | 0.551 |  |  |  |  |  |  |  |  |
|  | Vert high | 1.11 | 0.48 | 2.52 | 0.813 |  |  |  |  |  |  |  |  |
|  | **Residential address** |  |  |  | 0.859 |  |  |  |  |  |  |  |  |
|  | Village | Ref |  |  |  |  |  |  |  |  |  |  |  |
|  | City | 1.01 | 0.87 | 1.18 | 0.859 |  |  |  |  |  |  |  |  |
|  | **Marital status** |  |  |  | 0.412 |  |  |  |  |  |  |  |  |
|  | No | Ref |  |  |  |  |  |  |  |  |  |  |  |
|  | Yes | 0.77 | 0.41 | 1.44 | 0.412 |  |  |  |  |  |  |  |  |
|  | **Procreation status** |  |  |  | 0.128 |  |  |  |  |  |  |  |  |
|  | No | Ref |  |  |  |  |  |  |  |  |  |  |  |
|  | Yes | 0.64 | 0.36 | 1.14 | 0.128 |  |  |  |  |  |  |  |  |
|  | **Smoking and drinking consumption** |  |  |  | 0.218 |  |  |  |  |  |  |  |  |
|  | No | Ref |  |  |  |  |  |  |  |  |  |  |  |
|  | Smoking | 0.87 | 0.72 | 1.05 | 0.137 |  |  |  |  |  |  |  |  |
|  | Drinking | 1.30 | 0.87 | 1.93 | 0.198 |  |  |  |  |  |  |  |  |
|  | Both | 0.95 | 0.75 | 1.20 | 0.669 |  |  |  |  |  |  |  |  |
|  | **ASA score** |  |  |  | 0.102 |  |  |  |  |  |  |  |  |
|  | I | Ref |  |  |  |  |  |  |  |  |  |  |  |
|  | II | 1.09 | 0.94 | 1.28 | 0.256 |  |  |  |  |  |  |  |  |
|  | III-IV | 1.41 | 1.00 | 1.99 | **0.047** |  |  |  |  |  |  |  |  |
|  | **Abdominal surgery history** |  |  |  | 0.350 |  |  |  |  |  |  |  |  |
|  | No | Ref |  |  |  |  |  |  |  |  |  |  |  |
|  | Yes | 0.90 | 0.72 | 1.13 | 0.350 |  |  |  |  |  |  |  |  |
|  | **Intra-abdominal surgery history** |  |  |  | 0.881 |  |  |  |  |  |  |  |  |
|  | No | Ref |  |  |  |  |  |  |  |  |  |  |  |
|  | Yes | 1.02 | 0.77 | 1.35 | 0.881 |  |  |  |  |  |  |  |  |
|  | **Comorbidity** |  |  |  | 0.620 |  |  |  |  |  |  |  |  |
|  | No | Ref |  |  |  |  |  |  |  |  |  |  |  |
|  | Yes | 1.04 | 0.89 | 1.22 | 0.620 |  |  |  |  |  |  |  |  |
| Tumor's risk | **Tumor site** |  |  |  | **<0.001** |  |  |  | 0.252 |  |  |  |  |
|  | Lower | Ref |  |  |  | Ref |  |  |  |  |  |  |  |
|  | Middle | 1.25 | 1.02 | 1.53 | **0.029** | 1.14 | 0.92 | 1.40 | 0.233 |  |  |  |  |
|  | Upper | 1.09 | 0.90 | 1.32 | 0.403 | 1.02 | 0.84 | 1.24 | 0.838 |  |  |  |  |
|  | Overlapping lesion of stomach | 1.83 | 1.47 | 2.27 | **<0.001** | 1.24 | 0.98 | 1.57 | 0.073 |  |  |  |  |
|  | **pTNM stage** |  |  |  | **<0.001** |  |  |  | **<0.001** |  |  |  | **<0.001** |
|  | IIA | Ref |  |  |  | Ref |  |  |  | Ref |  |  |  |
|  | IIB | 1.30 | 0.91 | 1.86 | 0.149 | 1.27 | 0.88 | 1.82 | 0.200 | 1.28 | 0.89 | 1.84 | 0.181 |
|  | IIIA | 1.82 | 1.29 | 2.55 | **0.001** | 1.79 | 1.27 | 2.53 | **0.001** | 1.82 | 1.29 | 2.57 | **0.001** |
|  | IIIB | 3.02 | 2.24 | 4.08 | **<0.001** | 2.80 | 2.05 | 3.83 | **<0.001** | 2.92 | 2.14 | 3.97 | **<0.001** |
|  | IIIC | 5.44 | 4.07 | 7.28 | **<0.001** | 4.41 | 3.23 | 6.03 | **<0.001** | 4.76 | 3.51 | 6.47 | **<0.001** |
|  | **Tumor size, mm** |  |  |  | **<0.001** |  |  |  | **0.004** |  |  |  | **0.001** |
|  | <20 | Ref |  |  |  | Ref |  |  |  | Ref |  |  |  |
|  | 20-50 | 1.21 | 0.72 | 2.03 | 0.470 | 0.87 | 0.51 | 1.47 | 0.599 | 0.85 | 0.50 | 1.45 | 0.557 |
|  | >50 | 2.32 | 1.39 | 3.89 | **0.001** | 1.15 | 0.67 | 1.95 | 0.618 | 1.17 | 0.69 | 1.99 | 0.562 |
|  | **Lymphatic vessel infiltration** |  |  |  | **<0.001** |  |  |  | 0.213 |  |  |  |  |
|  | Negative | Ref |  |  |  | Ref |  |  |  |  |  |  |  |
|  | Positive | 1.34 | 1.15 | 1.55 | **<0.001** | 1.10 | 0.95 | 1.29 | 0.213 |  |  |  |  |
|  | **Pathological differentiation degree** |  |  |  | **0.002** |  |  |  | 0.397 |  |  |  |  |
|  | Differentiated | Ref |  |  |  | Ref |  |  |  |  |  |  |  |
|  | Undifferentiated | 0.75 | 0.63 | 0.91 | **0.002** | 0.92 | 0.76 | 1.12 | 0.397 |  |  |  |  |
|  | **Examined LNs, No.** |  |  |  | **<0.001** |  |  |  | **<0.001** |  |  |  | **<0.001** |
|  | >15 | Ref |  |  |  | Ref |  |  |  | Ref |  |  |  |
|  | ≤15 | 2.06 | 1.40 | 3.03 | **<0.001** | 2.52 | 1.69 | 3.75 | **<0.001** | 2.44 | 1.65 | 3.62 | **<0.001** |
|  | **Clavien-Dindo grade** |  |  |  | **0.001** |  |  |  | 0.085 |  |  |  | 0.082 |
|  | None | Ref |  |  |  | Ref |  |  |  | Ref |  |  |  |
|  | I-II | 1.35 | 1.10 | 1.65 | **0.005** | 1.22 | 0.99 | 1.51 | 0.067 | 1.22 | 0.99 | 1.50 | 0.068 |
|  | III-IV | 1.56 | 1.15 | 2.13 | **0.005** | 1.27 | 0.93 | 1.76 | 0.138 | 1.28 | 0.93 | 1.76 | 0.130 |
|  | **Chemotherapy** |  |  |  | **<0.001** |  |  |  | 0.074 |  |  |  | 0.075 |
|  | AC group | Ref |  |  |  | Ref |  |  |  | Ref |  |  |  |
|  | UAC group | 1.33 | 1.14 | 1.55 | **<0.001** | 1.16 | 0.99 | 1.37 | 0.074 | 1.16 | 0.99 | 1.37 | 0.075 |

AC: Acceptable chemotherapy, UAC: Unacceptable delay or missing chemotherapy, LN: lymph node

Supplementary Table 2. Changes in the risks for predicting patients with unacceptable chemotherapy

| Variables | | Period | | | | | |
| --- | --- | --- | --- | --- | --- | --- | --- |
|  |  | 2011 | 2012 | 2013 | 2014 | 2015 Jan-Apr | P value |
| Patients' risk | Gender(Female) | 24.9 | 24.2 | 25.1 | 27.4 | 30.7 | 0.671 |
|  | Age, year(≥65) | 43.4 | 40.5 | 41.7 | 36.6 | 42.0 | 0.445 |
|  | Medical insurance type(Self-paid)† | 13.0 | 11.4 | 9.3 | 7.5 | 4.5 | **0.043** |
|  | Social status(Very low)† | 3.2 | 1.3 | 0.0 | 0.0 | 4.5 | **<0.001** |
|  | ASA score(III-IV) | 4.3 | 3.6 | 5.2 | 3.6 | 5.7 | 0.708 |
|  | Abdominal surgery history(Yes) | 14.7 | 13.5 | 13.4 | 12.8 | 11.4 | 0.920 |
| Treatment risk | Clavien-Dindo grade(III-IV) | 4.6 | 4.2 | 6.7 | 4.5 | 4.5 | 0.549 |

Supplementary Table 3 The number of patients during different period of chemotherapy

| Variable | No. of patients | % |
| --- | --- | --- |
| No chemotherapy | 477 | 31.4 |
| <4 Weeks | 349 | 23.0 |
| 4-6 Weeks | 503 | 33.1 |
| 6-8 Weeks | 107 | 7.0 |
| 8-10 Weeks | 47 | 3.1 |
| 10-12 Weeks | 27 | 1.8 |
| >12 Weeks | 10 | 0.7 |
